# Differential trafficking of albumin and IgG facilitated by the neonatal Fc receptor in podocytes in vitro and in vivo

**DOI:** 10.1371/journal.pone.0209732

**Published:** 2019-02-27

**Authors:** James Dylewski, Evgenia Dobrinskikh, Linda Lewis, Pantipa Tonsawan, Makoto Miyazaki, Parmjit S. Jat, Judith Blaine

**Affiliations:** 1 Division of Renal Diseases and Hypertension, University of Colorado Denver, Aurora CO, United States of America; 2 Division of Nephrology, Denver Health Medical Center, Denver CO, United States of America; 3 Pulmonary Division, University of Colorado Denver, Aurora CO, United States of America; 4 Division of Nephrology, Department of Medicine, Khon Kaen University, Khon Kaen, Thailand; 5 MRC Prion Unit at University College London, Institute of Prion Diseases, London, United Kingdom; National Cancer Institute, UNITED STATES

## Abstract

Proteinuria is strongly associated with kidney disease progression but the mechanisms underlying podocyte handling of serum proteins such as albumin and IgG remain to be elucidated. We have previously shown that albumin and IgG are transcytosed by podocytes in vitro. In other epithelial cells, the neonatal Fc receptor (FcRn) is required to salvage albumin and IgG from the degradative pathway thereby allowing these proteins to be transcytosed or recycled. Here we directly examine the role of FcRn in albumin and IgG trafficking in podocytes by studying handling of these proteins in FcRn knockout (KO) podocytes in vitro and in a podocyte-specific FcRn knockout mice in vivo. In vitro, we find that knockout of FcRn leads to IgG accumulation in podocytes but does not alter albumin trafficking. Similarly, in vivo, podocyte-specific knockout of FcRn does not result in albumin accumulation in podocytes in vivo as measured by mean albumin fluorescence intensity whereas these mice demonstrate significant intraglomerular accumulation of IgG over time. In addition we find that podocyte-specific FcRn KO mice demonstrate mesangial expansion as they age and activation of mesangial cells as demonstrated by increased expression of α-smooth muscle actin. Taken together, these results suggest that trafficking pathways for albumin and IgG differ in podocytes and that sustained disruption of trafficking of plasma proteins alters glomerular structure.

## Introduction

Proteinuria is an independent marker of kidney disease progression and is widely used clinically as a biomarker of kidney dysfunction [1, 2]. Proteinuria is both a consequence of kidney damage and damages the glomerulus and tubules directly by increasing production of pro-inflammatory cytokines and promoting fibrosis [1, 3–5]. Both the glomerulus and the proximal tubules are involved in the renal handling of serum proteins but the molecular mechanisms remain to be fully elucidated. The primary barrier to filtration of large plasma proteins into the urine is the glomerular filtration barrier (GFB) which consists of three layers–a fenestrated endothelium, the glomerular basement membrane and the podocyte [6]. The podocyte is a specialized epithelial cell containing a large cell body and multiple processes which ramify to form smaller processes. Paracellular passage of large serum proteins is prevented by the slit diaphragm which extends between the foot processes of neighboring podocytes and precludes filtration of proteins ~ 70 kDa or larger. The precise amount of albumin filtered through the GFB is a contested topic [7, 8]. By even the most conservative estimates, ~ 4 g albumin a day transit the GFB [9]. The amount of IgG that traverses the glomerular filtration barrier is unknown.

Podocytes have been shown to take up albumin in vitro and in vivo [4, 10–12]. Using in vitro assays, we have previously shown that podocytes endocytose albumin and that most is transcytosed, with a smaller amount sent to the lysosome for degradation [13]. These findings have been confirmed in vivo using intravital multiphoton microscopy in rats [11]. In other epithelial cells, including those in the renal proximal tubule, the neonatal Fc receptor (FcRn) is required to prevent albumin and IgG from entering the degradative pathway, thereby allowing albumin to be recycled or transcytosed [14–17]. FcRn, has homology to major histocompatibility complex class I and binds albumin and IgG at pH 6–6.5 but has minimal affinity for these proteins at neutral pH [18]. Within the adult kidney, FcRn is expressed in podocytes, vascular endothelial cells and the proximal tubule [19]. The physiologic role of FcRn in albumin trafficking in podocytes is unknown.

Akilesh et al. demonstrated that the neonatal Fc receptor is required to prevent the intraglomerular accumulation of IgG in mice [20]. These studies were performed in global FcRn knockout (KO) mice which manifest hypoalbuminemia and hypogammaglobulinemia. Plasma levels of albumin and IgG are 50% [17] and 80–90% [21] lower respectively in global FcRn KO compared to wild type (WT) mice. Thus podocytes in the global KO are exposed to significantly less albumin and IgG than WT mice which might alter trafficking pathways.

Here we use in vitro assays and podocyte-specific FcRn knockout mice to directly examine the role of FcRn in albumin and IgG trafficking in podocytes. Creation of podocyte-specific FcRn KO mice allowed for the examination of intraglomerular trafficking of albumin and IgG in mice that have normal serum levels of these proteins, permitting direct assessment of FcRn mediated trafficking of albumin and IgG in podocytes.

## Materials and methods

### Generation of conditionally immortalized WT and FcRn KO podocytes

Podocytes were isolated from wild type or global FcRn KO mice as previously described [22]. Primary podocytes were immortalized using a thermosensitive SV40 T antigen as previously described [23]. Briefly, media containing viral particles was collected from the viral producer line plpcx SVtsa58 (kindly provided by Dr. Parmjit Jat) and applied to primary WT or FcRn KO podocytes. The plpcs SVtsa58 viral producer line encodes the thermolabile tsA58 LT antigen and G418 resistance. Podocytes were selected using G418. After selection, podocytes were allowed to replicate at 33°C. To induce differentiation, podocytes were placed at 37°C for 8–10 days. To verify expression of podocyte markers, podocytes were stained with podocin or WT1.

### In vitro trafficking assay

The in vitro albumin and IgG trafficking experiments were performed as previously described [13]. Briefly, WT or FcRn KO podocytes were loaded with 1.5 mg/ml FITC-human albumin or 1 mg/ml human IgG at 4°C (which permits binding and inhibits endocytosis) or 37°C (which permits endocytosis). Previous work has shown that mouse FcRn binds both human albumin and IgG at the concentrations used in these studies [24]. After loading, cells were washed well and incubated in Ringer solution (122.5 mM NaCl, 5.4 mM KCl, 1.2 mM CaCl_2_, 0.8 mM MgCl_2_, 0.8 mM Na_2_HPO_4_, 0.2 mM NaH_2_PO_4_, 5.5 mM glucose, and 10 mM HEPES; pH 7.4) at 4°C or 37°C in the presence or absence of 20 μM leupeptin (which inhibits lysosomal degradation). Cells and supernatant were either harvested immediately (t = 0) or at the designated time points. The supernatant was removed by evaporation under a vacuum and the remaining albumin or IgG resuspended in 35 μl RIPA buffer. The amount of albumin or IgG in the cellular or fraction was assessed by western blot analysis.

### Western blotting

Podocytes were harvested by scraping into RIPA buffer and protein concentrations were measured using the BCA assay (Pierce, Thermofisher Scientific, Waltham, MA). Samples were reduced using 10% β-mercaptoethanol. The Western blot procedure was performed as described in detail in [13, 25]. Briefly, cell lysates were run on 10% polyacrylamide gels and transferred onto nitrocellulose membranes (Bio-Rad, Hercules, CA). Subsequent blocking, antibody, and wash solutions were diluted in PBS-T (phosphate-buffered saline, 1% Triton-X 100). Membranes were blocked in 5% nonfat dry milk in PBS-T for 60 minutes and then incubated with primary antibody. The primary antibodies used were as follows: FITC (1:1,000; clone ZF2471–1900, Invitrogen; Carlsbad, CA), IgG (1:1000; GW20083F, Sigma-Aldrich, St. Louis, MO), actin (1:5,000; A1978, Sigma-Aldrich). Blots were washed and then incubated with horseradish peroxidase-conjugated secondary antibodies (1:10,000 dilution; Jackson ImmunoResearch, West Grove, PA). The antibody complexes were detected using enhanced chemiluminescence (Pierce) and Western blot images were captured using a photodocumentation system (UVP; Upland, CA).

### PCR

Total RNA was isolated using RNeasy Mini Kit (QIAGEN, Valencia, CA). cDNA was synthesized from total RNA (1 μg) with the High Capacity cDNA Reverse Transcriptase Kit (Invitrogen) which uses the random primer scheme. The primers used for FcRn are as follows: sense 5′-TGA CCT GTG CTG CTT TCT CCT-3′, antisense 5′-CAG CAA TGA CCA TGC GTG GAA-3′. Real-time PCR was performed with the use of the AppIied Biosystems Step One Plus Real-Time PCR System (Life Technologies, Carlsbad, CA). The expression of a target gene in relation to a reference gene was calculated using a comparative cycle threshold (Ct) method.

### Animals

Podocyte specific FcRn knockout mice were obtained by crossing FcRn floxed mice [26] (a kind gift of Dr. Sally Ward, UT Southwestern) with podocin-Cre mice (Jackson Labs, Bar Harbor, Maine). Genotype was determined by PCR. All experimental mice were homozygous for the floxed FcRn gene. Podocyte specific FcRn knockout mice (FcRn fl/fl;cre/+) were double transgenic resulting in no FcRn expression in podocytes. Control mice (FcRn fl/fl;+/+) were single transgenic (no Cre expression) resulting in unchanged FcRn expression in podocytes. Male mice were used for all experiments. For the aging studies the number of mice is as follows: 3 month old control or podocyte specific FcRn knockout, n = 3 animals per group; 6 month old control or podocyte specific FcRn knockout, n = 6 animals per group; 12 month old control or podocyte specific FcRn knockout, n = 6 animals per group. All procedures involving animals were performed using protocols approved by the Institutional Animal Care and Use Committee at the University of Colorado, Denver, protocol number 00085. Animals were euthanized using pentobarbital.

Urine albumin was measured using the Albuwell assay (Exocell), urine creatinine was measured using the assay and BUN was measured on an Alpha Wasserman auto analyzer. Serum albumin was measured by ELISA (Abcam) as was serum IgG (Affymetrix).

For the GFR measurements, urine was collected by placing mice in metabolic cages for 24 hours. Serum and urine creatinine concentrations were analyzed with HPLC-MS/MS (Applied Biosystems 3200 Qtrap). Creatinine and [^2^H3] creatinine were detected in multiple reaction monitoring mode, monitoring the transitions of the *m*/*z* from 114 to 44.2 and from 117 to 47.2, respectively [27]. Mice were aged 9–12 months. Four male control and 3 podocyte-specific FcRn KO mice were used.

### Immunofluorescence

Confocal microscopy images were acquired using Zeiss 780 laser-scanning confocal/multiphoton-excitation fluorescence microscope with a 34-Channel GaAsP QUASAR Detection Unit and non-descanned detectors for two-photon fluorescence (Zeiss, Thornwood, NY). The imaging settings were initially set to maximize the signal-to-noise ratio, avoid saturation and ensure minimal contributions from tubular autoflluorescence. The settings were kept constant between different samples. Images were obtained with a Zeiss C-Apochromat 40x/1.2NA Korr FCS M27 water-immersion lens objective. The illumination for imaging was provided by a 30mW Argon Laser using excitation at 488 nm, HeNe 5mW (633 nm) and 1mW (543 nm). Image processing was performed using Zeiss ZEN 2012 software. Images were analyzed in Image J software (NIH, Bethesda, Maryland). Fluorescence intensity of albumin or IgG was normalized to glomerular area. 20–25 glomeruli were analyzed per mouse.

Preparation of cells for fixed cell images was performed as described in detail in [13]. Podocytes were fixed in 4% paraformaldehyde in phosphate-buffered saline (PBS) with 0.5% Triton X-100 (20 min; room temp), washed, blocked with 10% normal serum and labeled with primary antibodies. Primary antibodies were as follows: Podocin (1:200, P0372, Sigma-Aldrich), WT-1 (1: 200, sc-192, Santa Cruz), FcRn (1: 100, sc-66892, Santa Cruz). Cells were subsequently washed and labeled with the appropriate conjugated secondary antibodies (Alexa Fluor 488, Alexa Fluor 568; Invitrogen). F-actin was concurrently stained with Alexa-Phalloidin 633 (Invitrogen).

For the in vivo immunolocalization studies, the kidneys were cleared of blood by perfusion of phosphate-buffered saline (PBS) and then fixed by perfusion with 4% paraformaldehyde (Electron Microscopy Sciences; Hatfield, PA) in PBS (pH 7.4). The kidneys were then removed, immersed in 4% paraformaldehyde for 24hr, infused with 5% (2 hr), 10% (2 hr) and 25% (overnight) sucrose, frozen in liquid nitrogen and cryosectioned (3 μm). Kidney sections were blocked (10% normal goat serum in PBS) and incubated overnight at 4°C with primary antibody: IgG (1:250; GW20083F, Sigma-Aldrich), albumin (1:250; ab106582, Abcam, Cambridge, UK), a-SMA (1:250; 1A4, Sigma-Aldrich). After washing, the sections were incubated (60 min, room temperature) with appropriate mix of Alexa 488-conjugated goat anti-chicken IgG (1:500; Invitrogen) and Alexa 633-conjugated phalloidin (1:200; Invitrogen). Sections were then washed with PBS and mounted in Fluromount-G (Thermo Fisher Scientific, Waltham, MA).

### Histology

3 μm sections were cut from paraffin embedded tissue and stained using the periodic acid Schiff reagent. Analysis of glomerular and mesangial area was performed using NDP.view 2 (Hamamatsu, Hamamatsu City, Japan). 20–30 glomeruli were analyzed per mouse.

### Data analysis

Data are presented as means ± SE. Statistical analysis was performed using *t*-tests for two groups and one-way analysis of variance for three or more groups, using Prism software (GraphPad, San Diego, CA). Tukey's post hoc test was applied to the ANOVA data. Values were considered statistically significant when *p* < 0.05.

## Results

### Establishing WT and FcRn KO podocyte cell lines

**Fig 1:**
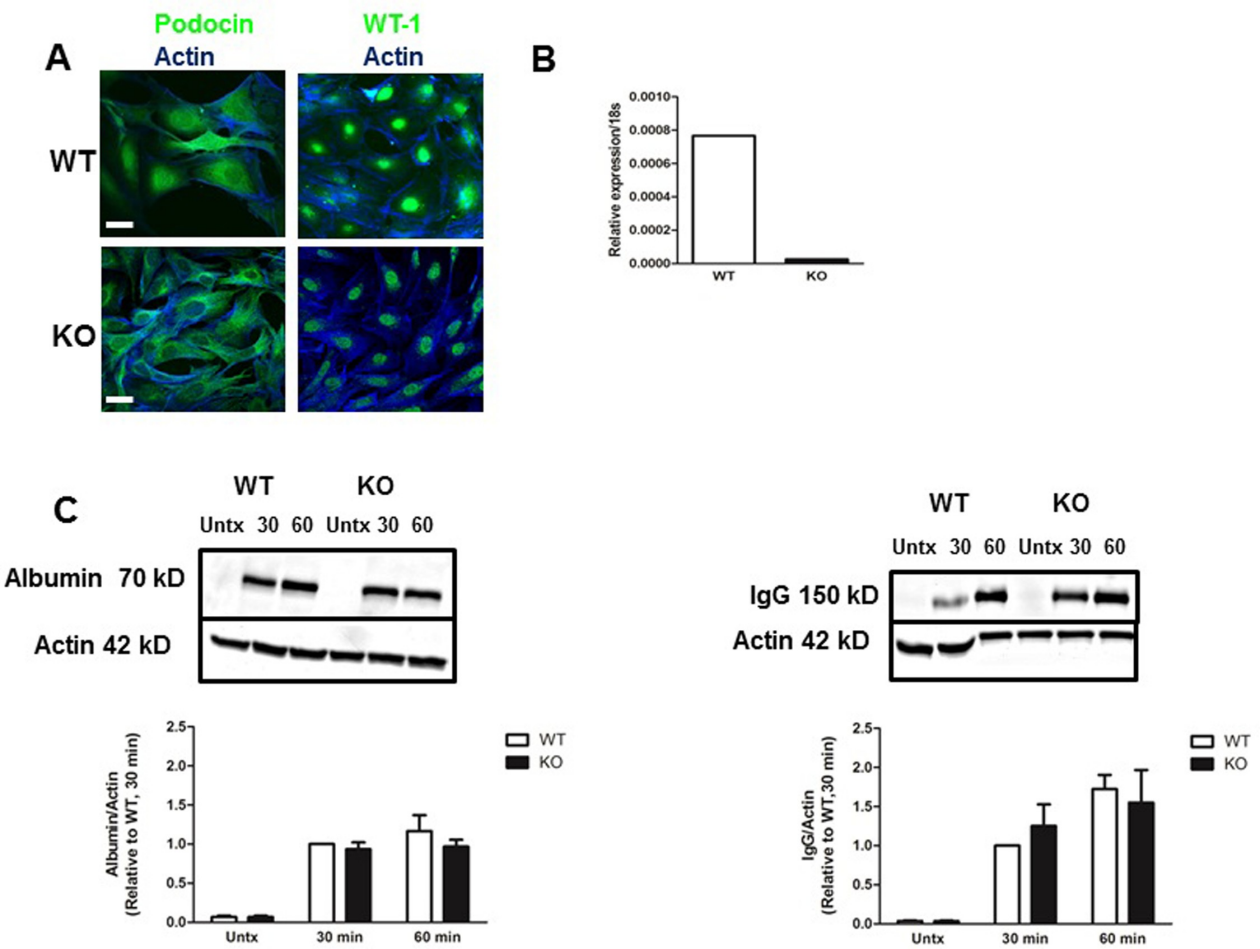
Characterization of FcRn knockout podocytes. *A*, Conditionally immortalized wild type (WT) and FcRn knockout (KO) podocytes express the podocyte markers podocin and Wilms Tumor 1 (WT1). Scale bar 20 μm. *B*, FcRn KO podocytes have minimal expression of FcRn mRNA. *C*, There was no significant differences in uptake of FITC-albumin or IgG in wild type versus FcRn KO podocytes. n = 3 experiments.

In order to directly investigate the role of FcRn in albumin and IgG trafficking in podocytes, we isolated podocytes from WT and global FcRn KO mice and created conditionally immortalized cell lines by transforming primary podocytes with the thermosensitive SV40 T antigen [23]. Differentiated WT and FcRn KO podocytes expressed the podocyte markers podocin and Wilms tumor 1 (WT1) (Fig 1A) and FcRn KO podocytes had minimal FcRn mRNA as assessed by qPCR (Fig 1B). FcRn knockout did not impair albumin or IgG uptake in podocytes (Fig 1C).

### FcRn KO impairs IgG trafficking but not albumin trafficking in vitro

**Fig 2:**
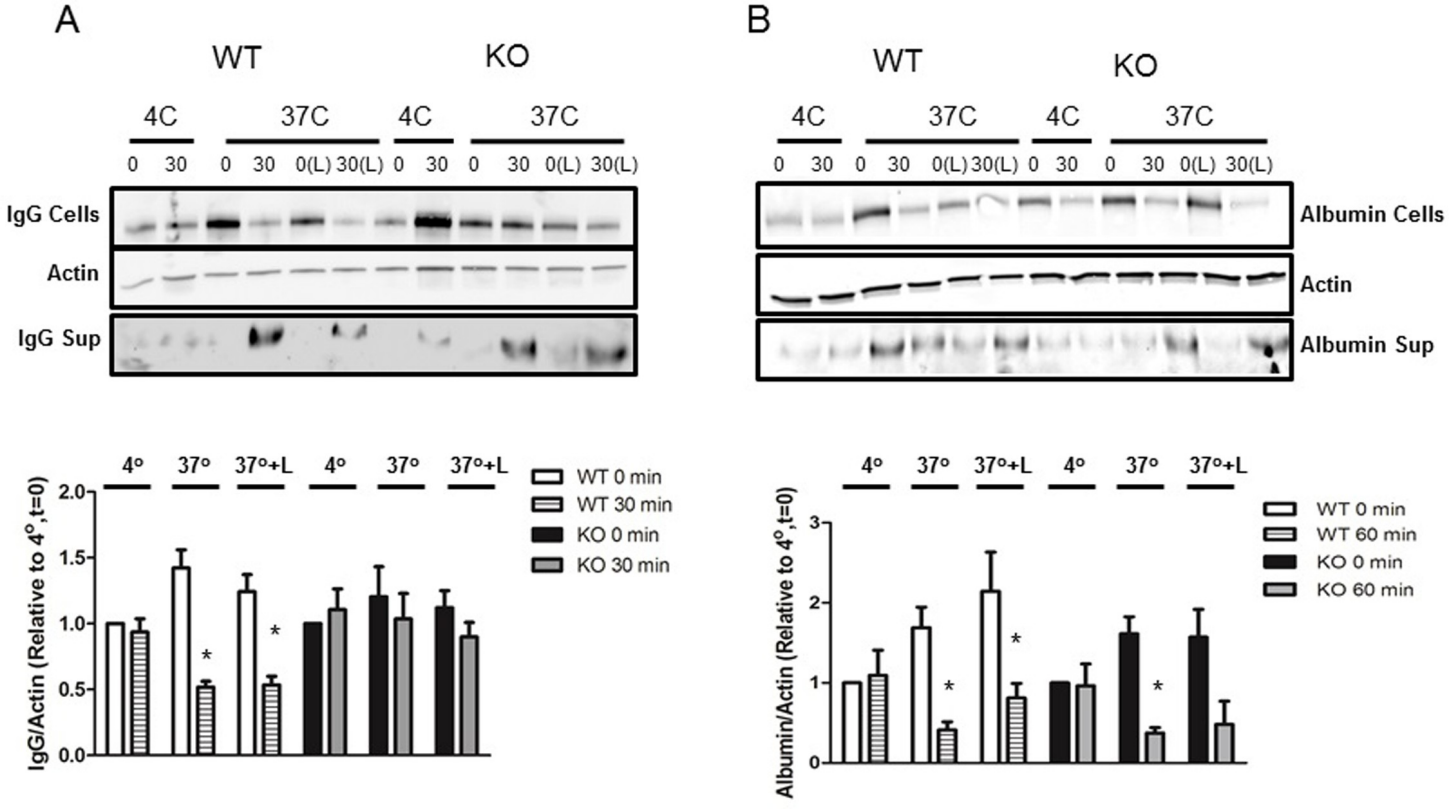
FcRn KO impaired albumin but not IgG transcytosis in podocytes in vitro. *A*, Cultured podocytes from FcRn KO mice demonstrate impaired IgG transcytosis. There is significantly less monomeric IgG in the cellular fraction in WT podocytes 30 min after loading with IgG, whereas the amount of monomeric IgG in the cellular fraction in KO podocytes is comparable to that at t = 0. Inhibition of lysosomal degradation does not alter the amount of monomeric IgG present in the cellular fraction suggesting that IgG is not sent to the lysosome. *, p = 0.0017 compared to the same condition at t = 0; n = 10 experiments. Time (0, 30) is in minutes; L = leupeptin. *B*, In contrast, FcRn KO has no effect on albumin transcytosis in cultured podocytes. There is significantly less albumin in the cellular fraction in both WT and KO podocytes 60 minutes after loading with albumin. *, p < 0.0001 compared to the same condition at t = 0; n = 8 experiments. Time (0, 60) is in minutes; L = leupeptin.

To examine IgG and albumin trafficking in vitro, differentiated WT and FcRn KO podocytes were loaded with albumin or IgG at 4°C (a temperature that allows surface binding of albumin or IgG but inhibits endocytosis) or 37°C (permits endocytosis), washed well after loading and harvested immediately or at the times indicated in Fig 2. Leupeptin, a lysosomal enzyme inhibitor, was also used to examine the effects of blocking lysosomal degradation on albumin and IgG trafficking in WT and FcRn KO podocytes. In WT podocytes loaded with IgG at 37°C, there was a decrease in the amount of IgG remaining in the cells after 30 minutes incubation in Ringer solution (Fig 2A). Inhibition of lysosomal degradation with leupeptin did not significantly increase the amount of IgG remaining in WT podocytes after 30 minutes incubation in Ringer solution, suggesting that monomeric IgG is not trafficked to the lysosome. In contrast to WT podocytes, the amount of IgG remaining intracellularly in FcRn KO podocytes 30 minutes after loading with IgG was not significantly decreased, suggesting impairment in IgG transcytosis in the KO (Fig 2A).

Albumin trafficking in WT and FcRn KO podocytes was examined by loading podocytes with FITC-labeled albumin, washing the podocytes very well and then assessing how much FITC-albumin remained in the podocytes immediately after washing or after 60 minutes in Ringer solution ± leupeptin to inhibit lysosomal degradation. Podocytes were assessed after 60 min as initial studies demonstrated that albumin is trafficked more slowly than IgG. As shown in Fig 2B, in both WT and FcRn KO podocytes loaded with FITC-albumin at 37°C there was a significant decrease in the amount of albumin remaining in the cells 60 minutes after incubation in Ringer solution. Thus, knockout of FcRn did not impair albumin trafficking in podocytes in vitro. In both WT and KO podocytes, there was a trend towards increased albumin accumulation in leupeptin treated cells at t = 60 minutes, 37°C but this was not significant.

### Podocyte-specific FcRn KO mice

**Fig 3:**
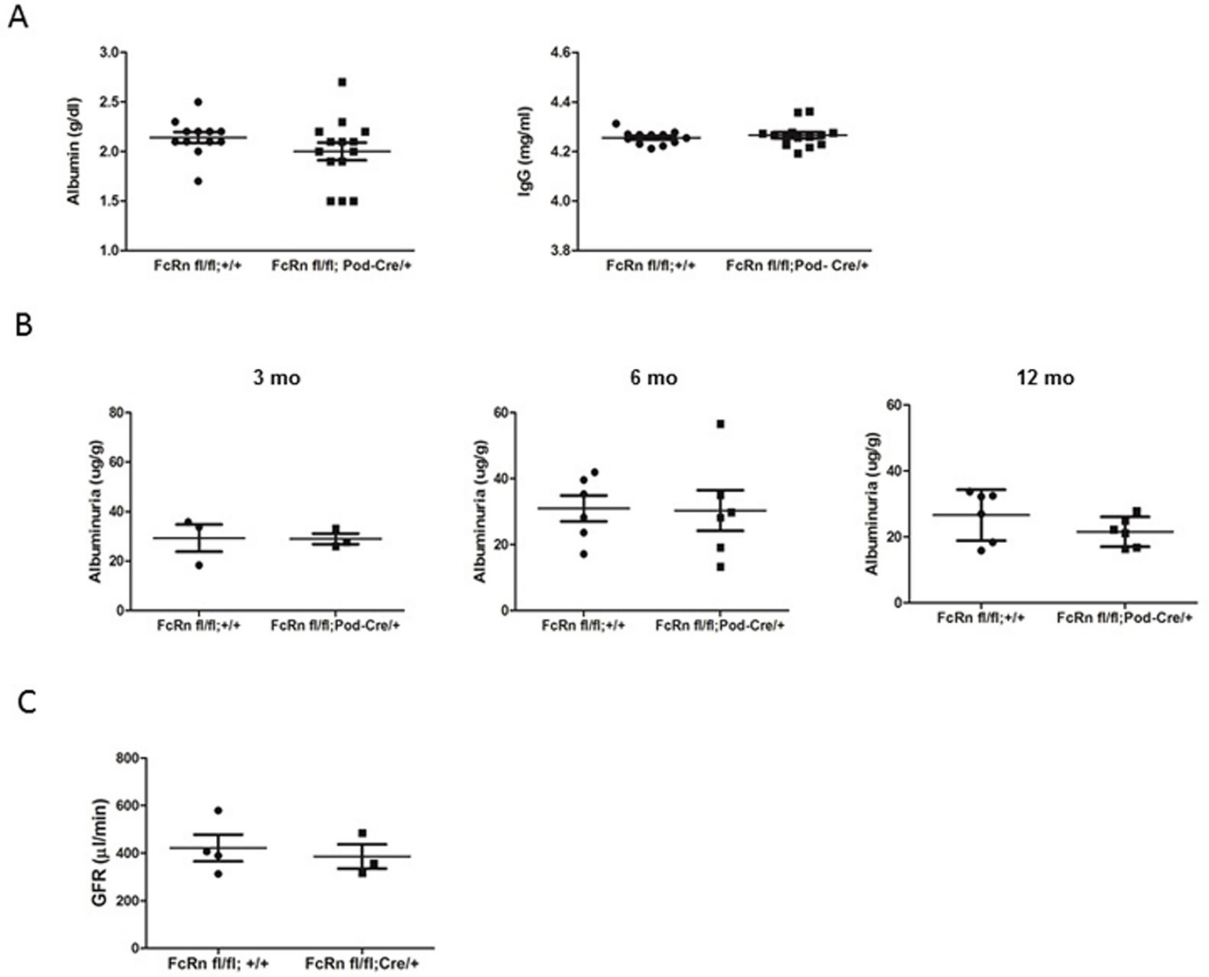
Functional parameters in podocyte-specific FcRn knock-out mice. *A*, There were no differences in serum albumin or IgG levels between podocyte-specific FcRn KO (FcRn fl/fl; Pod-Cre/+) and control (FcRn fl/fl;+/+) mice (n = 14 KO and n = 12 control mice). *B*, Podocyte-specific FcRn KO and control mice had minimal albuminuria at 3 months and no significant increase in albuminuria with age (n = 3 control and 3 KO mice at 3 months, 6 KO and 6 control mice at 6 months and 6 KO and 6 control mice at 12 months). C., There was no significant difference in GFR measurements in 9–12 month old control or podocyte-specific FcRn KO mice (n = 4 control and 3 KO mice).

In order to determine whether the differential trafficking of albumin and IgG in podocytes lacking FcRn occurred in vivo, we generated podocyte-specific FcRn KO (FcRn fl/fl;Podocin-Cre/+) mice by crossing podocin-Cre mice with FcRn floxed mice (Fig 3). FcRn floxed mice lacking the Cre transgene (FcRn fl/fl;+/+) served as littermate controls. FcRn fl/fl;Podocin-Cre/+ mice had similar serum levels of albumin and IgG compared to controls (Fig 3A). There was no difference in the urinary albumin to creatinine ratio in podocyte-specific FcRn KO mice versus control at 3, 6 and 12 months (Fig 3B). There was also no difference in estimated glomerular filtration rate (eGFR) in 9–12 month control or podocyte specific FcRn KO mice (Fig 3C).

### Podocyte-specific KO of FcRn results in IgG accumulation in the glomerulus

**Fig 4:**
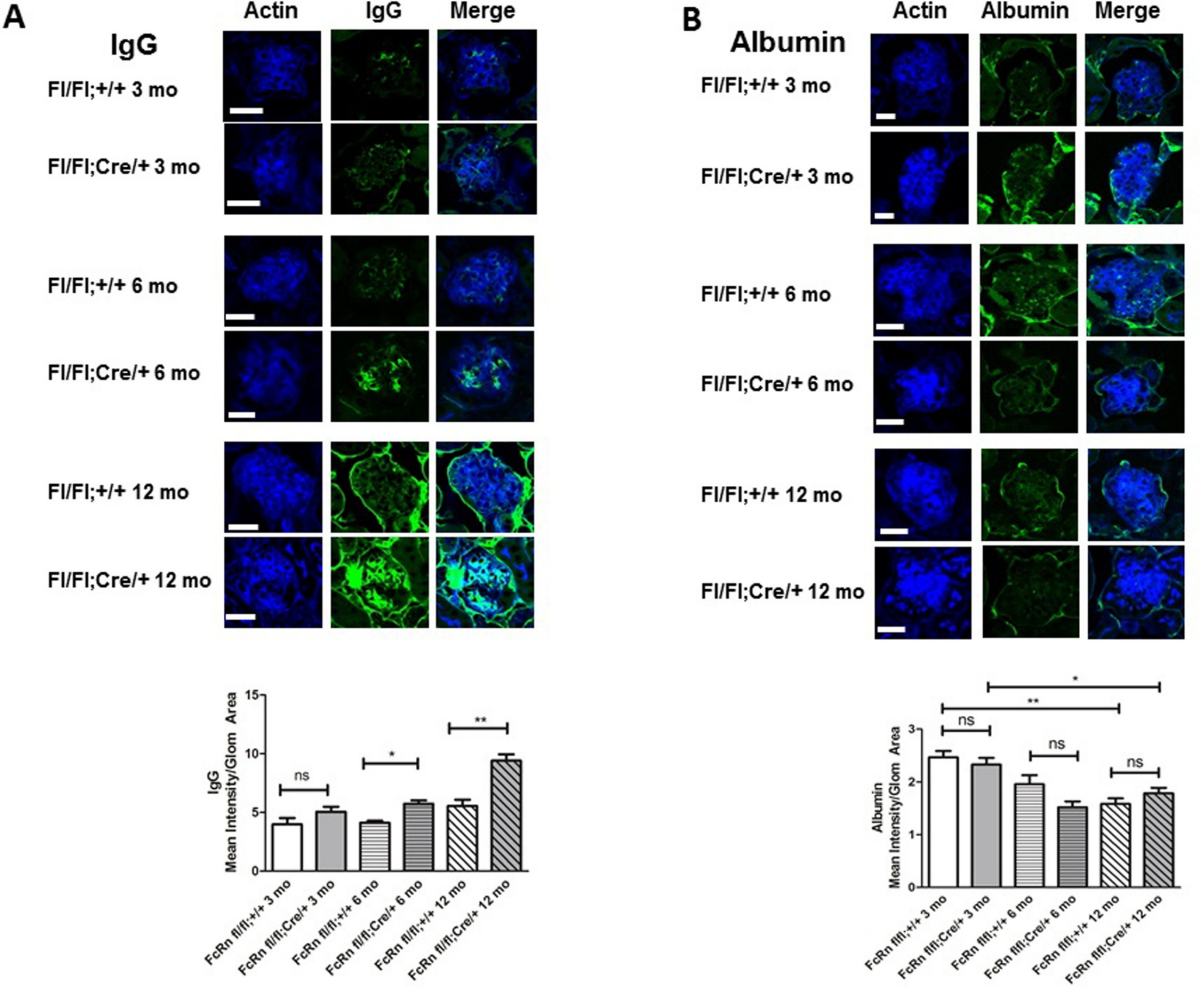
Podocyte-specific FcRn KO resulted in a significant increase in intraglomerular IgG accumulation, but no change in albumin accumulation. *A*, Intraglomerular IgG: At 3 months of age, there was no significant difference in IgG accumulation between podocyte specific FcRn KO (fl/fl; cre/+) and control mice (fl/fll;+/+). (n = 3 mice per group). By 6 months of age there was a statistically significant increase in intraglomerular IgG accumulation in podocyte-specific FcRn KO (fl/fl;cre/+) compared to controls (fl/fl;+/+) (n = 6 control and 6 KO mice, * p < 0.05). Intraglomerular IgG accumulation was even more significantly increased by 12 months of age in the podocyte-specific FcRn KO (n = 6 control and 6 KO mice, ** p < 0.0001). Scale bar 20 μm. *B*, Intraglomerular Albumin: Albumin accumulation in control and podocyte-specific FcRn KO mouse glomeruli was minimal and was not significantly different between control and KO animals at 3, 6 or 12 months. By 12 months of age, both control and podocyte-specific FcRn KO mice had significantly less intraglomerular albumin than 3 month old control or KO animals, * p < 0.01, ** p < 0.0001. Scale bar 20 μm. NS = not significant. Number of mice per group was the same as in *A*.

We used immunofluorescence staining to examine whether lack of FcRn in podocytes results in albumin or IgG accumulation in the glomerulus as mice age. There was no significant difference in IgG accumulation in the glomeruli of podocyte-specific FcRn KO versus control mice at 3 months of age (mean IgG fluorescence/glomerular area 3.9 ± 0.5 vs 5.1 ± 0.4, p = NS; Fig 4A). By 6 months of age, podocyte-specific FcRn KO mice had a significant increase in glomerular IgG compared to controls (5.7 ± 0.3 vs 4.1 ± 0.2, p < 0.05). At 12 months of age there was a statistically significant increase in the amount of IgG present in the glomerulus in podocyte-specific FcRn KO compared to controls (9.4 ± 0.5 vs 5.6 ± 0.5, p < 0.0001).

### Podocyte-specific KO of FcRn results in minimal intraglomerular albumin accumulation

When mean albumin intensity per glomerulus was measured, there was no significant difference in albumin accumulation in the glomeruli of 3 month, 6 month or 12 month old versus podocyte-specific FcRn KO versus control mice (Fig 4B). Interestingly, there was a significant time dependent decrease in intraglomerular albumin accumulation with 12 month old control and podocyte-specific FcRn KO mice exhibiting a significant decrease in albumin accumulation within the glomerulus compared to the respective 3 month old mice (mean albumin fluorescence/glomerular area for 12 month versus 3 month old mice 1.5 ± 0.1 vs 2.5 ± 0.1 for controls, p < 0.0001 and 1.8 ± 0.1 vs 2.3 ± 0.1 for KO, p < 0.01). The lack of albumin detection within the glomerulus was not due to an inability to stain for albumin as albumin within the blood vessels surround the glomerulus was readily detected (Fig 4B).

**Fig 5:**
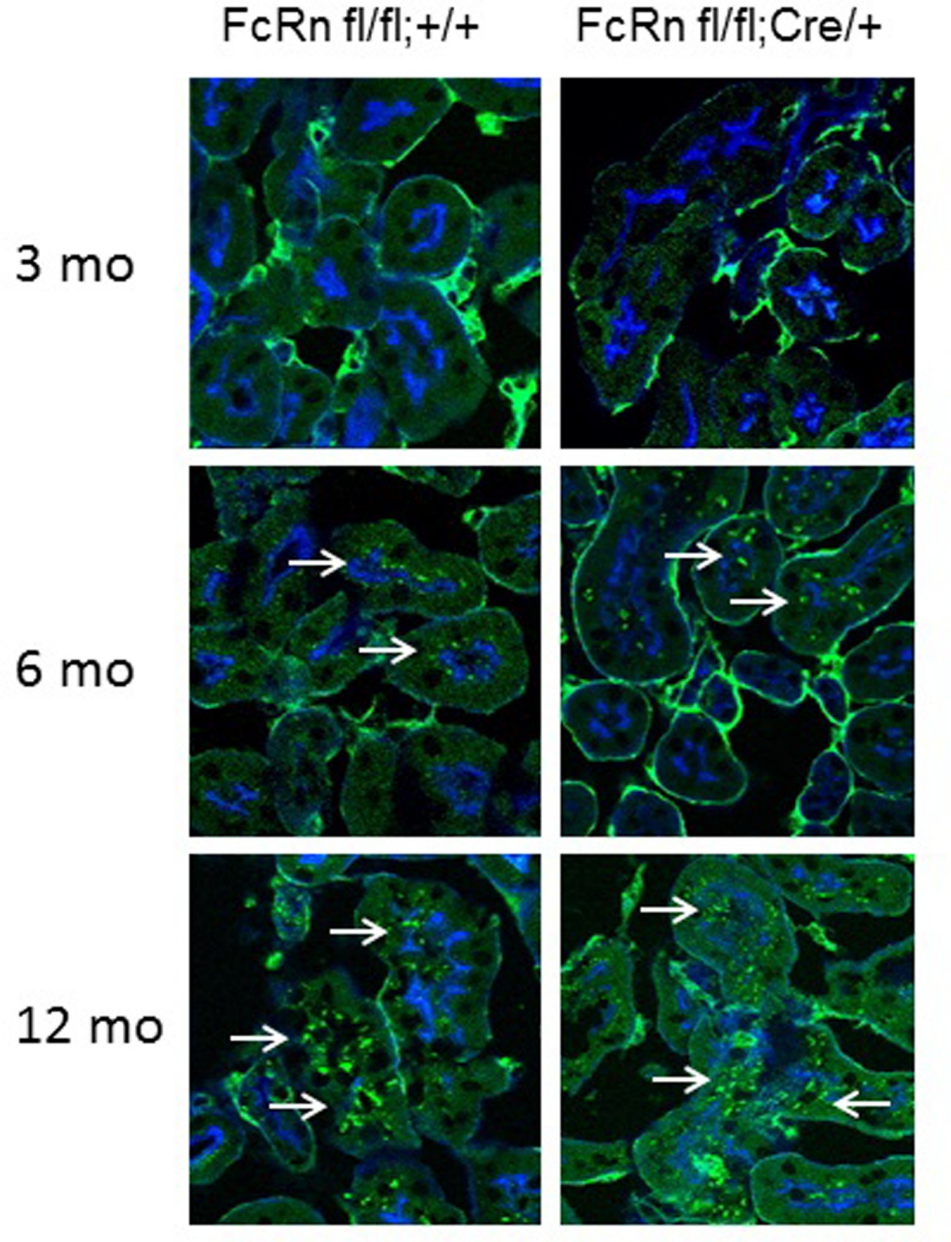
Albumin accumulates in control and podocyte-specific FcRn knockout mice with age. Albumin vesicles (green) are not seen in control or podocyte-specific FcRn knockout at 3 months of age. By 6 months of age, albumin is seen in tubules in both control and KO mice (arrows) with more prominent vesicles seen by 12 months in both control and KO animals.

The final concentration of albumin in the urine has been shown to be dependent on both passage of albumin through the glomerular filtration barrier and proximal tubular uptake of albumin [7, 10, 13, 28, 29]. Since podocyte specific KO of FcRn did not result in intraglomerular accumulation of albumin over time and KO mice demonstrated no increase in albuminuria with age, we examined whether there were differences in the tubular accumulation of albumin in control and podocyte specific FcRn KO animals. We found that at 3 months of age there was minimal accumulation of albumin in the tubules of both control and podocyte-specific FcRn KO mice. By 6 months of age, both control and KO mice had albumin accumulation within some tubules which was further increased by 12 months of age (Fig 5).

### Podocyte-specific KO of FcRn leads to increased mesangial:Glomerular area ratio

**Fig 6:**
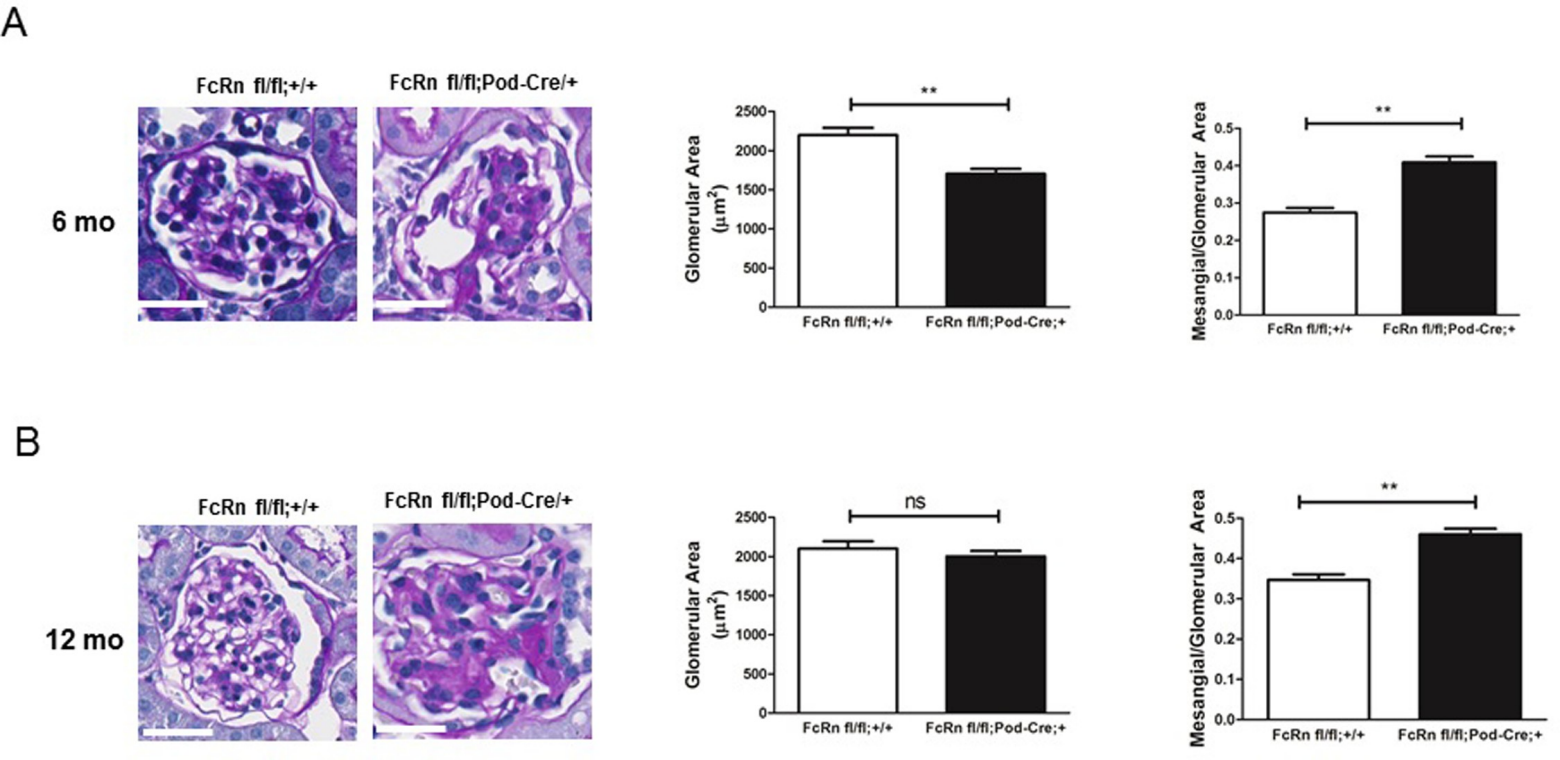
Podocyte-specific FcRn KO resulted in mesangial expansion as mice aged. *A*, 6 month old podocyte-specific FcRn KO mice had a decrease in mean glomerular area (**, p < 0.0001) and an increase in mesangial/glomerular area (**, p < 0.0001 compared to controls). Scale bar 20 μm. *B*, By 12 months of age, mean glomerular area was similar in podocyte-specific FcRn KO and control mice but the podocyte-specific KO manifested a further increase in glomerular/mesangial area (**, p < 0.0001 compared to controls). Scale bar 20 μm.

**Fig 7:**
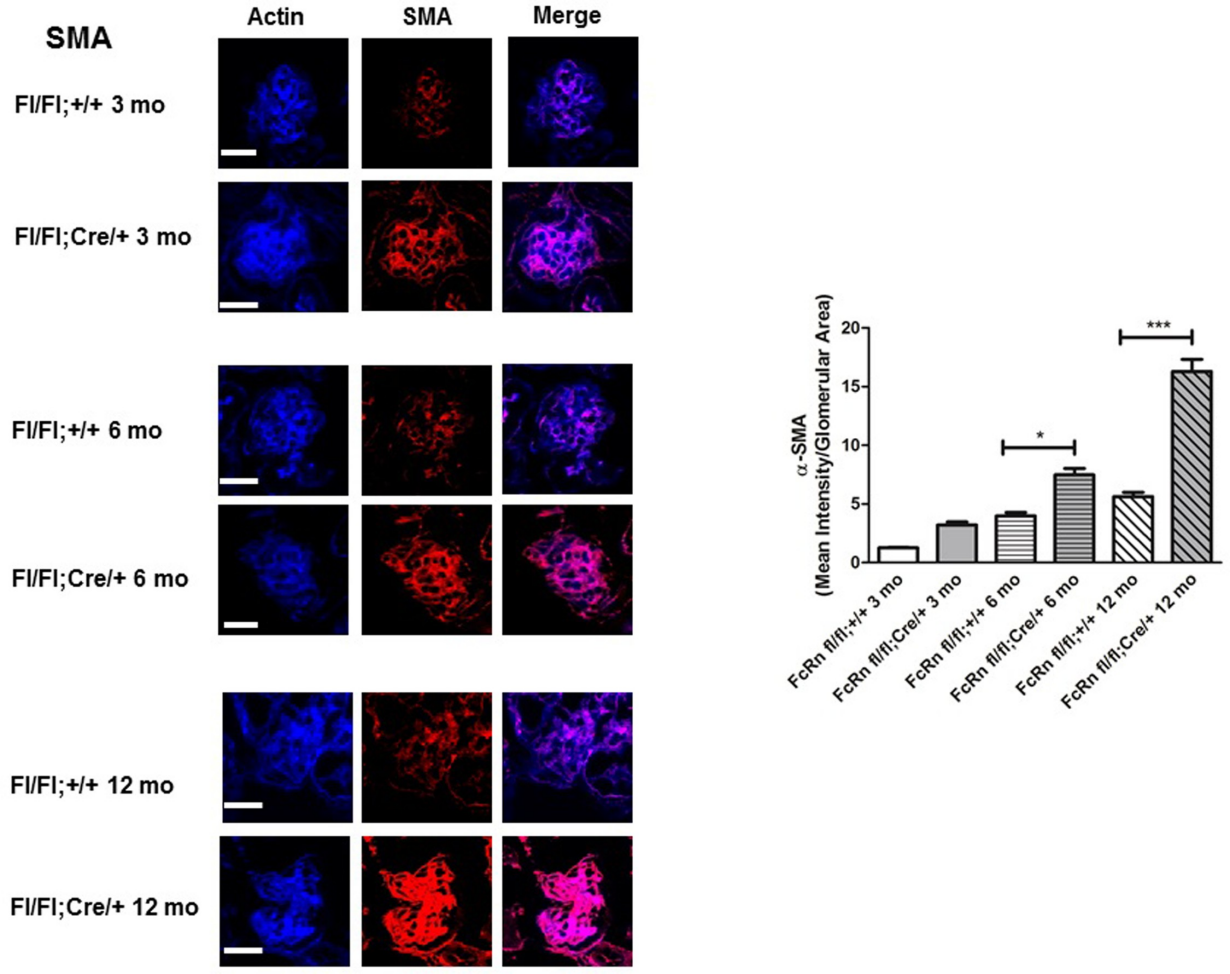
Podocyte-specific FcRn KO resulted in increased glomerular expression of α-smooth muscle actin. Intraglomerular expression of α-smooth muscle actin (α-SMA) was significantly increased in 6 month podocyte-specific FcRn KO (fl/fl;Cre/+) mice compared to controls (fl/fl;+/) (*, p < 0.01, n = 6 control and 6 KO mice). The increase in α-SMA expression was even more pronounced by 12 months of age (**, p < 0.0001, n = 6 control and 6 KO mice). Scale bar: 20 μm.

By 6 months of age, podocyte-specific FcRn KO mice had a significant decrease in glomerular area compared to control mice (1703 ± 62.7 μm^2^ 2198 ± 92.9 μm^2^, p < 0.0001) and a significant increase in the mesangial to glomerular area (0.41 ± 0.01 vs 0.27 ± 0.01, p < 0.0001; Fig 6A). By 12 months of age, there was a further significant increase in the mesangial/glomerular area in the podocyte-specific FcRn KO compared to controls (0.46 ± 0.01 vs 0.35 ± 0.01, p < 0.0001), with a resultant increase in the glomerular area in the KO to close to that of control (Fig 6B). To further examine the mesangial expansion seen in the podocyte-specific FcRn KO mice, we examined the glomerular expression of α-smooth muscle actin (α-SMA), a marker of activated mesangial cells [30]. We found an increase in glomerular α-SMA actin expression by 3 months in podocyte-specific FcRn KO versus control which was statistically significant by 6 and 12 month months (α-SMA intensity/glomerular area for 3 month KO vs control 3.2 ± 0.3 vs 1.2 ± 0.1; for 6 month KO vs control 7.5 ± 0.5 vs 4.0 ± 0.3, p < 0.01; for 12 month KO versus control 16.3 ± 1.0 vs 5.6 ± 0.3, p < 0.0001, Fig 7). While older podocyte specific FcRn KO mice demonstrated mesangial expansion, there was no significant difference in eGFR in 9–12 month control or KO mice (Fig 3C).

## Discussion

Proteinuria is a common clinical marker of kidney damage and is strongly associated with progression of kidney disease [2, 31]. The mechanisms underlying renal handling of serum proteins such as albumin and IgG remain to be fully elucidated. Human and animal studies have shown podocyte vacuolization in proteinuric kidney diseases and albumin and IgG have been shown to colocalize with podocyte vacuoles [5, 10, 32–37]. Previously, we have shown that cultured podocytes endocytose albumin and that the majority of endocytosed albumin is transcytosed. Here we extend our findings to an in vivo model and also examine podocyte handling of IgG. To our knowledge, this is the first systematic concurrent examination of podocyte albumin and IgG trafficking in podocytes.

In renal proximal tubular cells, FcRn is required for salvaging albumin and IgG from the degradative pathway and transcytosing these proteins from the apical cell surface to the basolateral side [16]. Others have shown that injection of an anti-FcRn antibody reduces proteinuria in nephrotic rats [34] and that global FcRn KO mice accumulate IgG within the glomerulus by 6 months of age [20]. Our study, however, is the first to directly demonstrate the role of FcRn in albumin and IgG handling in cell culture and in an in vivo model in which only podocytes lack FcRn. Our studies demonstrate that albumin and IgG are differentially handled by podocytes in vitro and in vivo. Lack of FcRn in cultured podocytes did not impair albumin handling acutely. In vivo, lack of FcRn did not result in significant intraglomerular accumulation of albumin. Interestingly, mice had significantly less intraglomerular albumin accumulation at 12 months compared to 3 months in both podocyte-specific FcRn KO animals and controls. Lack of intraglomerular albumin accumulation in podocyte-specific FcRn KO mice was not due to technical issues with staining as albumin could be seen in the peritubular and periglomerular capillaries.

Intravital imaging has provided evidence for passage of albumin through the glomerular filtration barrier [7, 8, 29, 38], although the amount of albumin to transverse the GFB is widely debated. Our study provides further evidence for passage of albumin through the GFB as both unmanipulated control and podocyte-specific FcRn knockout mice demonstrate albumin accumulation within renal tubules over time. The precise mechanisms for albumin handling in podocytes remain to be fully determined. Gianesello et al. have shown that megalin, cubilin, amionless and the ClCl-5 channel are involved in the uptake of low doses of albumin in cultured human podocytes [12] and we have shown that albumin endocytosis in cultured podocytes is also dependent on caveolin [13]. Schiessl et al. have shown that in vivo, angiotensin II dependent passage of albumin through the glomerular filtration barrier is dependent on megalin [39]. In addition, we have found that in cultured human podocytes, endocytosed albumin is degraded primarily in the lysosome [25]. In the proximal tubule, FcRn is required to divert endocytosed albumin from the lysosmal degradative pathway. In the present study, however, we find that in vitro, FcRn is not required for albumin transcytosis through the podocyte nor does lack of FcRn in vivo lead to intraglomerular accumulation of albumin. The lack of albumin accumulation in podocytes both in vivo and in vitro suggests that podocytes possess another non-FcRn dependent pathway for handling any serum albumin that passes through the GFB.

Knockout of FcRn in podocytes led acutely to intracellular accumulation of IgG in vitro and accumulation of IgG in vivo which became significant by 6 months of age. Lack of FcRn, however, did not completely abrogate IgG transcytosis in cultured podocytes as evidenced by the fact that some IgG appeared in the supernatant in FcRn KO cells after loading podocytes with IgG and incubating in IgG free solution, suggesting the existence of an additional non-FcRn dependent pathway for IgG handling.

Akilesh et al. found increased IgG accumulation in the glomeruli of global FcRn KO mice compared to wild type at 6 months of age [20]. There are several important differences between our study and that of Akilesh et al. We used podocyte-specific FcRn KO mice which have normal circulating levels of albumin and IgG whereas Akilesh et al. used global FcRn KO mice which have serum albumin levels that are 50% lower and serum IgG levels that are 80–90% lower than those of wild type animals. In addition, we performed systematic quantitation of albumin and IgG staining in podocyte-specific FcRn KO mice or controls as mice aged using confocal microscopy whereas Akilesh et al. used epifluorescence microscopy on wild type or global FcRn KO mice at a single time point (6 months) and did not perform any quantitation.

An interesting finding of the present study is that podocyte-specific knockout of FcRn leads to mesangial expansion in the KO mice as they age as well as increased expression of α-smooth muscle actin within the mesangial regions of the glomerulus, suggesting activation of mesangial cells with intraglomerular IgG accumulation. Upregulation of a-smooth muscle actin has been linked to progression of fibrosis [40], suggesting that dysregulated serum protein trafficking in podocytes might contribute to CKD progression. The mechanisms underlying how knockout of a trafficking protein in a podocyte can lead to an expansion of mesangial area and increased expression of α-SMA remain to be further investigated.

In summary we have directly examined the role of FcRn in albumin and IgG trafficking in poodcytes and found that FcRn-mediated trafficking of these proteins differs. Podocyte-specific FcRn knockout impairs glomerular handling of IgG whereas albumin handling is not affected. Differences in intra podocyte trafficking of these proteins may reflect differences in their biologic functions. Albumin often serves as a carrier for different molecules such as lipids whereas IgG is a component of the immune system. Our findings suggest that intra-podocyte trafficking pathways are complex and that disruption of normal trafficking pathways in podocytes is deleterious.

## Supporting information

S1 Fig Uncropped images for western blots in Fig 1C. (TIF)

S2 Fig Uncropped images for western blots in Fig 2A and 2B. (TIF)

## References

[1] Abbate M, Zoja C, Remuzzi G. How Does Proteinuria Cause Progressive Renal Damage? Journal of the American Society of Nephrology. 2006;17(11):2974–84. 10.1681/ASN.2006040377 17035611

[2] Hemmelgarn BR, Manns BJ, Lloyd A, James MT, Klarenbach S, Quinn RR, et al Relation Between Kidney Function, Proteinuria, and Adverse Outcomes. JAMA: The Journal of the American Medical Association. 2010;303(5):423–9. 10.1001/jama.2010.39 20124537

[3] Abbate M, Zoja C, Morigi M, Rottoli D, Angioletti S, Tomasoni S, et al Transforming Growth Factor-β1 Is Up-Regulated by Podocytes in Response to Excess Intraglomerular Passage of Proteins: A Central Pathway in Progressive Glomerulosclerosis. The American journal of pathology. 2002;161(6):2179–93. 10.1016/S0002-9440(10)64495-1 12466133 PMC1850904

[4] Okamura K, Dummer P, Kopp J, Qiu L, Levi M, Faubel S, et al Endocytosis of Albumin by Podocytes Elicits an Inflammatory Response and Induces Apoptotic Cell Death. PloS one. 2013;8(1):e54817 10.1371/journal.pone.0054817 23382978 PMC3557279

[5] Morigi M, Buelli S, Angioletti S, Zanchi C, Longaretti L, Zoja C, et al In Response to Protein Load Podocytes Reorganize Cytoskeleton and Modulate Endothelin-1 Gene: Implication for Permselective Dysfunction of Chronic Nephropathies. The American journal of pathology. 2005;166(5):1309–20. 10.1016/S0002-9440(10)62350-4 15855633 PMC1606387

[6] Scott RP, Quaggin SE. Review series: The cell biology of renal filtration. The Journal of cell biology. 2015;209(2):199–210. Epub 2015/04/29. 10.1083/jcb.201410017 25918223 PMC4411276

[7] Russo LM, Sandoval RM, McKee M, Osicka TM, Collins AB, Brown D, et al The normal kidney filters nephrotic levels of albumin retrieved by proximal tubule cells: Retrieval is disrupted in nephrotic states. Kidney international. 2007;71(6):504–13. http://www.nature.com/ki/journal/v71/n6/suppinfo/5002041s1.html. 10.1038/sj.ki.5002041 17228368

[8] Peti-Peterdi J. Independent two-photon measurements of albumin GSC give low values. American Journal of Physiology—Renal Physiology. 2009;296(6):F1255–F7. 10.1152/ajprenal.00144.2009 19297453 PMC2692442

[9] Castrop H, Schiessl IM. Novel routes of albumin passage across the glomerular filtration barrier. Acta physiologica (Oxford, England). 2016 Epub 2016/07/28. 10.1111/apha.12760 . 27452481

[10] Eyre J, Ioannou K, Grubb BD, Saleem MA, Mathieson PW, Brunskill NJ, et al Statin-sensitive endocytosis of albumin by glomerular podocytes. American journal of physiology Renal physiology. 2007;292(2):F674–81. 10.1152/ajprenal.00272.2006 17032937

[11] Schiessl IM, Hammer A, Kattler V, Gess B, Theilig F, Witzgall R, et al Intravital Imaging Reveals Angiotensin II-Induced Transcytosis of Albumin by Podocytes. Journal of the American Society of Nephrology: JASN. 2015 Epub 2015/06/28. 10.1681/asn.2014111125 . 26116357 PMC4769192

[12] Gianesello L, Priante G, Ceol M, Radu CM, Saleem MA, Simioni P, et al Albumin uptake in human podocytes: a possible role for the cubilin-amnionless (CUBAM) complex. Sci Rep. 2017;7(1):13705 Epub 2017/10/24. 10.1038/s41598-017-13789-z 29057905 PMC5651885

[13] Dobrinskikh E, Okamura K, Kopp JB, Doctor RB, Blaine J. Human podocytes perform polarized, caveolae-dependent albumin endocytosis. American journal of physiology Renal physiology. 2014;306(9):F941–51. Epub 2014/02/28. 10.1152/ajprenal.00532.2013 ; PubMed Central PMCID: PMCPmc4010685. 24573386 PMC4010685

[14] Andersen JT, Sandlie I. The Versatile MHC Class I-related FcRn Protects IgG and Albumin from Degradation: Implications for Development of New Diagnostics and Therapeutics. Drug Metabolism and Pharmacokinetics. 2009;24(4):318–32. 19745559 10.2133/dmpk.24.318

[15] Kim J, Bronson CL, Hayton WL, Radmacher MD, Roopenian DC, Robinson JM, et al Albumin turnover: FcRn-mediated recycling saves as much albumin from degradation as the liver produces. American journal of physiology Gastrointestinal and liver physiology. 2006;290(2):G352–60. Epub 2005/10/08. 10.1152/ajpgi.00286.2005 . 16210471

[16] Tenten V, Menzel S, Kunter U, Sicking E-M, van Roeyen CRC, Sanden SK, et al Albumin Is Recycled from the Primary Urine by Tubular Transcytosis. Journal of the American Society of Nephrology. 2013 10.1681/asn.2013010018 23970123 PMC3839546

[17] Chaudhury C, Mehnaz S, Robinson JM, Hayton WL, Pearl DK, Roopenian DC, et al The Major Histocompatibility Complex–related Fc Receptor for IgG (FcRn) Binds Albumin and Prolongs Its Lifespan. The Journal of experimental medicine. 2003;197(3):315–22. 10.1084/jem.20021829 12566415 PMC2193842

[18] Kuo T, Baker K, Yoshida M, Qiao S-W, Aveson V, Lencer W, et al Neonatal Fc Receptor: From Immunity to Therapeutics. Journal of clinical immunology. 2010;30(6):777–89. 10.1007/s10875-010-9468-4 20886282 PMC2970823

[19] Dickinson BL. Unraveling the immunopathogenesis of glomerular disease. Clinical Immunology. 2016;169:89–97. 10.1016/j.clim.2016.06.011 27373970

[20] Akilesh S, Huber TB, Wu H, Wang G, Hartleben Br, Kopp JB, et al Podocytes use FcRn to clear IgG from the glomerular basement membrane. Proceedings of the National Academy of Sciences. 2008;105(3):967–72. 10.1073/pnas.0711515105 18198272 PMC2242706

[21] Roopenian DC, Christianson GJ, Sproule TJ, Brown AC, Akilesh S, Jung N, et al The MHC Class I-Like IgG Receptor Controls Perinatal IgG Transport, IgG Homeostasis, and Fate of IgG-Fc-Coupled Drugs. The Journal of Immunology. 2003;170(7):3528–33. 12646614 10.4049/jimmunol.170.7.3528

[22] Dobrinskikh E, Lewis L, Brian Doctor R, Okamura K, Lee MG, Altmann C, et al Shank2 Regulates Renal Albumin Endocytosis. Physiological reports. 2015;3(9). Epub 2015/09/04. 10.14814/phy2.12510 ; PubMed Central PMCID: PMCPmc4600376. 26333830 PMC4600376

[23] Jat PS, Sharp PA. Cell lines established by a temperature-sensitive simian virus 40 large-T-antigen gene are growth restricted at the nonpermissive temperature. Molecular and cellular biology. 1989;9(4):1672–81. Epub 1989/04/01. ; PubMed Central PMCID: PMCPmc362586. 2542774 10.1128/mcb.9.4.1672-1681.1989 PMC362586

[24] Andersen JT, Daba MB, Berntzen G, Michaelsen TE, Sandlie I. Cross-species binding analyses of mouse and human neonatal Fc receptor show dramatic differences in immunoglobulin G and albumin binding. The Journal of biological chemistry. 2010;285(7):4826–36. Epub 2009/12/19. 10.1074/jbc.M109.081828 20018855 PMC2836088

[25] Carson JM, Okamura K, Wakashin H, McFann K, Dobrinskikh E, Kopp JB, et al Podocytes degrade endocytosed albumin primarily in lysosomes. PloS one. 2014;9(6):e99771 Epub 2014/06/14. 10.1371/journal.pone.0099771 ; PubMed Central PMCID: PMCPmc4055698. 24924335 PMC4055698

[26] Montoyo HP, Vaccaro C, Hafner M, Ober RJ, Mueller W, Ward ES. Conditional deletion of the MHC class I-related receptor FcRn reveals the sites of IgG homeostasis in mice. Proceedings of the National Academy of Sciences. 2009;106(8):2788–93. 10.1073/pnas.0810796106 19188594 PMC2650344

[27] Masuda M, Miyazaki-Anzai S, Keenan AL, Shiozaki Y, Okamura K, Chick WS, et al Activating transcription factor-4 promotes mineralization in vascular smooth muscle cells. JCI insight. 2016;1(18):e88646 Epub 2016/11/05. 10.1172/jci.insight.88646 27812542 PMC5085604

[28] Cui S, Verroust PJ, Moestrup SK, Christensen EI. Megalin/gp330 mediates uptake of albumin in renal proximal tubule. American Journal of Physiology—Renal Physiology. 1996;271(4):F900–F7. 10.1152/ajprenal.1996.271.4.F900 8898021

[29] Sandoval RM, Wagner MC, Patel M, Campos-Bilderback SB, Rhodes GJ, Wang E, et al Multiple Factors Influence Glomerular Albumin Permeability in Rats. Journal of the American Society of Nephrology. 2012;23(3):447–57. 10.1681/ASN.2011070666 22223875 PMC3294301

[30] Johnson RJ, Iida H, Alpers CE, Majesky MW, Schwartz SM, Pritzi P, et al Expression of smooth muscle cell phenotype by rat mesangial cells in immune complex nephritis. Alpha-smooth muscle actin is a marker of mesangial cell proliferation. The Journal of clinical investigation. 1991;87(3):847–58. 10.1172/JCI115089 . 1671868 PMC329873

[31] Gansevoort RT, Matsushita K, van der Velde M, Astor BC, Woodward M, Levey AS, et al Lower estimated GFR and higher albuminuria are associated with adverse kidney outcomes. A collaborative meta-analysis of general and high-risk population cohorts. Kidney international. 2011;80(1):93–104. Epub 2011/02/04. 10.1038/ki.2010.531 ; PubMed Central PMCID: PMCPmc3959732. 21289597 PMC3959732

[32] Yoshikawa N IH, Akamatsu R, Hazikano H, Okada S, Matsuo T. Glomerular podocyte vacuolation in focal segmental glomerulosclerosis. Arch Pathol Lab Med 1986;110(5):394–8. 3754422

[33] Kinugasa S, Tojo A, Sakai T, Fujita T. Silver-enhanced immunogold scanning electron microscopy using vibratome sections of rat kidneys: detection of albumin filtration and reabsorption. Medical Molecular Morphology. 2010;43(4):218–25. 10.1007/s00795-010-0500-9 21267698

[34] Kinugasa S, Tojo A, Sakai T, Tsumura H, Takahashi M, Hirata Y, et al Selective albuminuria via podocyte albumin transport in puromycin nephrotic rats is attenuated by an inhibitor of NADPH oxidase. Kidney international. 2011;80(12):1328–38. 10.1038/ki.2011.282 21849973

[35] Tojo A, Onozato M, Kitiyakara C, Kinugasa S, Fukuda S, Sakai T, et al Glomerular albumin filtration through podocyte cell body in puromycin aminonucleoside nephrotic rat. Medical Molecular Morphology. 2008;41(2):92–8. 10.1007/s00795-008-0397-8 18592163

[36] Farquhar MG, Palade GE. Segregation of ferritin in glomerular protein absorption droplets. The Journal of biophysical and biochemical cytology. 1960;7:297–304. Epub 1960/04/01. 13821609 10.1083/jcb.7.2.297 PMC2224798

[37] Kerjaschki D, Miettinen A, Farquhar MG. Initial events in the formation of immune deposits in passive Heymann nephritis. gp330-anti-gp330 immune complexes form in epithelial coated pits and rapidly become attached to the glomerular basement membrane. The Journal of experimental medicine. 1987;166(1):109–28. Epub 1987/07/01. 2885390 10.1084/jem.166.1.109 PMC2188651

[38] Schiessl IM, Castrop H. Angiotensin II AT2 receptor activation attenuates AT1 receptor-induced increases in the glomerular filtration of albumin: a multiphoton microscopy study. American journal of physiology Renal physiology. 2013;305(8):F1189–200. Epub 2013/08/16. 10.1152/ajprenal.00377.2013 . 23946289

[39] Castrop H, Schießl IM. Novel routes of albumin passage across the glomerular filtration barrier. Acta Physiologica. 2017;219(3):546–55. 10.1111/apha.12760 27452481

[40] Liu Y. Epithelial to mesenchymal transition in renal fibrogenesis: pathologic significance, molecular mechanism, and therapeutic intervention. Journal of the American Society of Nephrology: JASN. 2004;15(1):1–12. Epub 2003/12/25. . 14694152 10.1097/01.asn.0000106015.29070.e7

