# Supplementary figures and images for: Differential trafficking of albumin and IgG facilitated by the neonatal Fc receptor in podocytes in vitro and in vivo
Source: PLoS One. 2019 Feb 27;14(2):e0209732. doi: 10.1371/journal.pone.0209732 (PMC6392300; doi:10.1371/journal.pone.0209732)

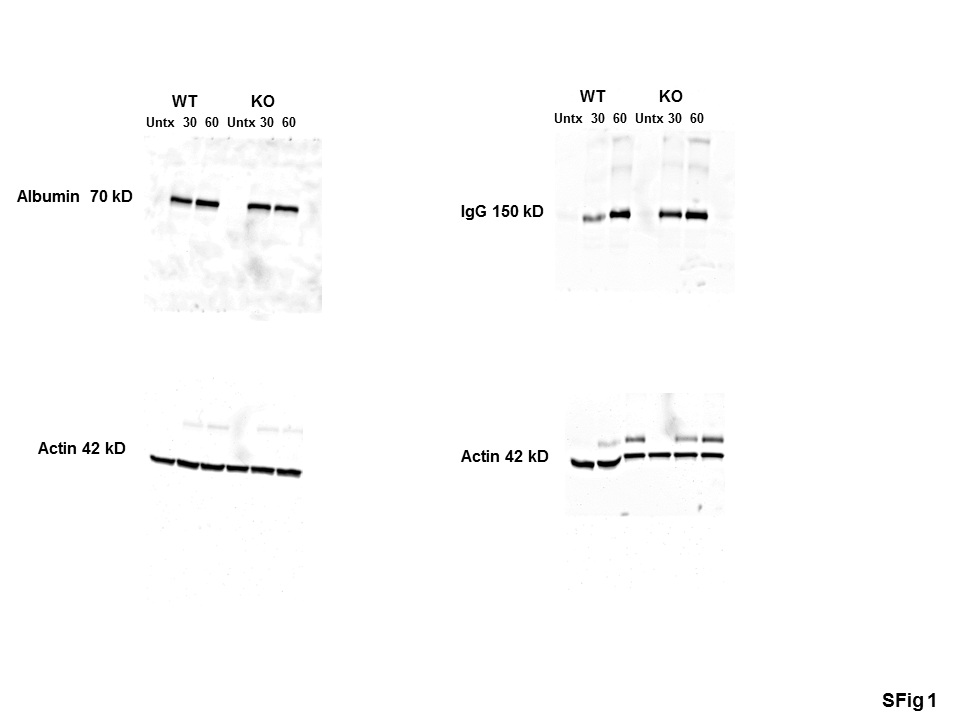

Supplement: S1 Fig — (TIF) [file pone.0209732.s001.tif]

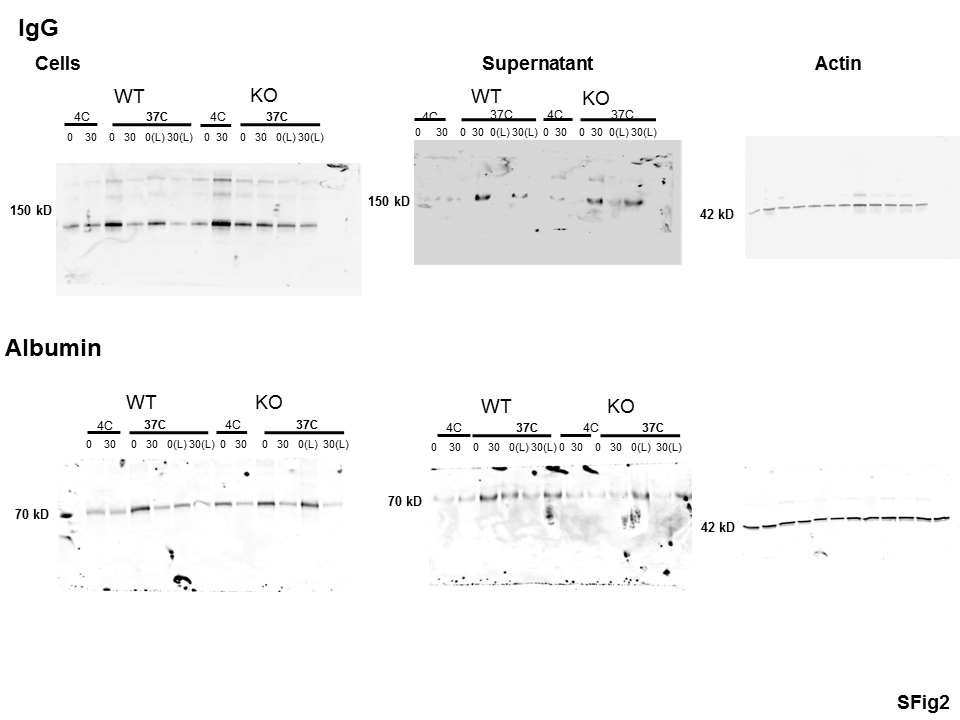

Supplement: S2 Fig — (TIF) [file pone.0209732.s002.tif]
